# Biomechanical evaluation of monosegmental pedicle instrumentation in a calf spine model and the role of fractured vertebrae in screw stability

**DOI:** 10.1186/s12917-016-0677-9

**Published:** 2016-03-18

**Authors:** Fuxin Wei, Zhiyu Zhou, Le Wang, Shaoyu Liu, Rui Zhong, Xizhe Liu, Shangbin Cui, Ximin Pan, Manman Gao, Yajing Zhao

**Affiliations:** Department of Spine Surgery, the First Affiliated Hospital and Orthopedic Research, Institute of Sun Yat-sen University, Guangzhou, China; The medical school of Shenzhen University, Shenzhen, China; Department of Radiology, the First Affiliated Hospital of Sun Yat-sen University, Guangzhou, China; The medical school of Sun Yat-sen University, Guangzhou, China

## Abstract

**Background:**

Monsegmental pedicle instrumentation (MSPI) has been used to treat thoracolumbar fractures. However, there are few reports about the biomechanical characteristics of MSPI compared with traditional short-segment pedicle instrumentation (SSPI) in management of unstable thoracolumbar fractures, and the influence of vertebral fracture on screw stability is still unclear.

**Methods:**

This study was to compare the immediate stability between MSPI and SSPI in management of unstable L1 fracture, and to evaluate the role of fractured vertebrae in screw stability. Two studies were performed: in the first study, sixteen fresh calf spines (T11-L3) were divided into two groups, in which unstable fractures at L1 were produced and then instrumented with MSPI or SSPI respectively. The range of motion (ROM) and lax zone (LZ) of specimens were evaluated with pure moment of 6 Nm loaded. The second study measured and compared the pullout strength of screws inserted in to 16 intact and fractured vertebrae of calf spines (L1-3) respectively. The correlation of pullout strength with load sharing classification (LSC) of fractured vertebrae was analyzed.

**Results:**

No significant difference in the ROM and LZ of the destabilized segments after fixation between MSPI and SSPI, except in axial rotation of ROM (*P* < 0.05). After fatigue cyclic loading, the MSPI showed a significant increase of ROM during lateral bending and axial rotation (*P* < 0.05); however, there were no significant differences in the LZ during all loading models between groups (*P* > 0.05). The mean pullout strength of pedicle screws in fractured vertebrae decreased by 13.7 %, compared with that of intact vertebrae (*P* > 0.05), and had a low correlation with LSC of the fractured vertebrae (*r* = 0.293, *P* > 0.05).

**Conclusions:**

MSPI can provide effective immediate stability for management of unstable thoracolumbar fractures; however, it has less fatigue resistance during lateral bending and axial rotation compared with SSPI. LSC score of fractured vertebrae is not a major influence on the pullout strength of screws.

## Background

Most thoracolumbar fractures are stable injuries that can be treated nonoperatively [1]. However, unstable fractures with retropulsed bone fragments and canal compromise usually warrant surgical intervention. The introduction of transpedicular screws by Roy-Camille and Demeulenaer, followed by the development of the internal fixator by Dick et al. [2], has made the short-segment pedicle screw instrumentation (SSPI) a popular method [3, 4]. After the development of the load-sharing classification (LSC) [5], more and more authors believe that on the condition of no severe anterior column defect, treatment of thoracolumbar burst fractures with SSPI can achieve clinical success [6, 7].

Saving motion segments by limiting the number of the fusion segments has been as a fundamental principle of spinal surgery [8]. Some authors have tried using monosegmental pedicle instrumentation (MSPI) with placement of pedicle screws directly into the fractured and normal vertebral body adjacent to the fractured endplate to treat thoracolumbar fractures, especially in cases of flexion distraction injuries [3, 9], and yielded good clinical results [10, 11]. Although the biomechanical properties of various similar approaches have been reported in the literature, there are few reports about the biomechanical characteristics of MSPI and whether the monosegmental pedicle instrumentation can provide immediate stability equivalent to that provided by SSPI is uncertain. In addition, complications of screw loosening and correction loss were not uncommon when performing MSPI in management of thoracolumbar fractures [11], especially for those combined with a load-sharing score of more than 6 points, which was reported in our previous clinical study [12]. To our knowledge, the contribution of the fractured vertebrae to screw stability and the relationship between the screw stability and the load sharing classification of fractured vertebrae has also not been well documented.

To quantify the biomechanical characteristics of MSPI in management of unstable thoracolumbar fractures, we performed two studies: the first study compared the immediate stability of the two pedicle instrumentation methods (MSPI and SSPI) in management of unstable thoracolumbar fractures; the second study investigated the role of fractured vertebrae on the screw stability and analyzed the correlation of the screw stability with load sharing classification of the fractured vertebrae.

## Methods

The study protocol was reviewed and approved by the institutional review board and ethics committee of the First Affiliated Hospital of our university (No. 2013–204).

### Study 1

#### Specimen preparation

Sixteen fresh frozen calf spines (T11-L3) were harvested with the age at time of death ranged from 6 to 8 weeks, which has been found to be the age at which calf spines best mimic the adult human spine [13]. In preparation, standard anteroposterior and lateral plain films were obtained and inspected to rule out pathologic abnormalities, and then surrounding soft tissue and muscle were dissected with care to preserve bone, discs and spinal ligaments. Both ends of the specimens were embedded with polymethylmethacrylate(PMMA) in a square aluminum mounting cast. The specimens were wrapped in saline-soaked gauze, kept in double plastic bags, and stored frozen at −30 °C. Before testing, each specimen was thawed at room temperature in a humidity-controlled environment for 12–18 h. To avoid influence on the biomechanical behavior by autolysis and air exposure, all specimens were kept moist during the tests by spraying saline onto them. Handling and storage of cadaver material in this manner, routinely used in vitro biomechanical investigation, does not alter the material characteristics of the bone and soft tissues [14]. All specimens were randomly allocated into two groups: the MSPI and SSPI groups.

#### Testing protocol

**Fig. 1:**
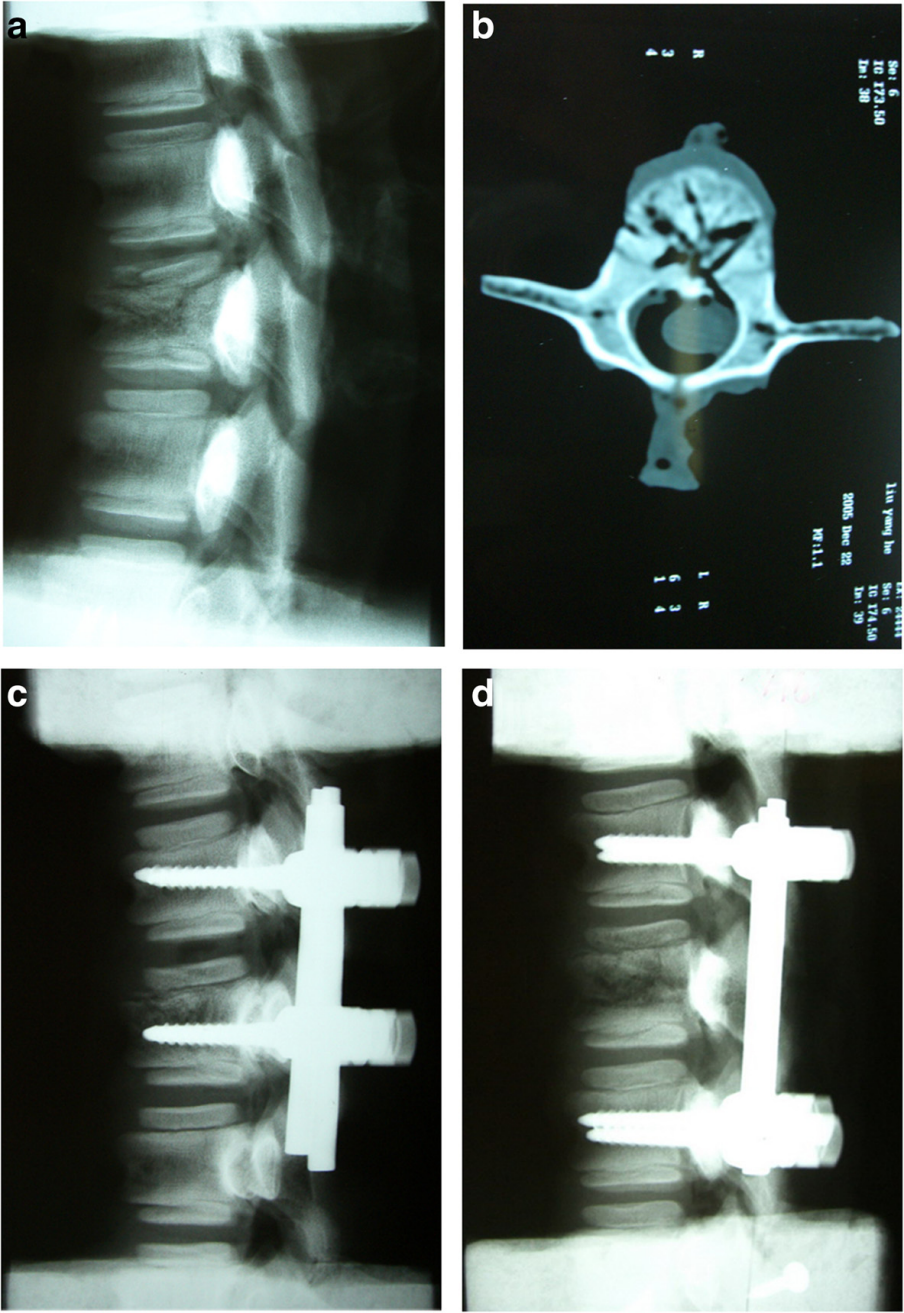
Radiographs showing post-injury specimens and that after pedicle instrumentation. **a** Lateral radiographs showing vertebral fractures at L1 level. **b** Axial computed tomography (CT) scan showing vertebral fractures and disruption of posterior edge of the vertebral body. **c** Monosegmental pedicle instrumentation (MSPI) with placement of pedicle screws directly into the fractured and normal vertebral body adjacent to the fractured endplate. **d** Traditional short-segment pedicle instrumentation (SSPI) with placement of pedicle screws into the upper and lower vertebral bodies adjacent to the fractured vertebral body

Flexibility of specimens was tested in 4 conditions (Table 1). Unstable 3-column fractures at the L-1 level were created by using a servohydraulic mechanical testing machine (MTS858 Bionix machine, MTS system Inc., Minneapolis, MN). To create a consistent injury pattern, a preinjury was created in each specimen, with reference to the methods reported previously [15, 16]. Minimal osteotomies were made with an osteotome at the upper endplate, and a 2-mm drill bit was used to create eight holes parallel and oblique to the intervertebral discs in the anterior cortex of the L1 vertebral body, extending to the posterior edge of the vertebral body. Then, the specimens were mounted in the MTS spinal fixture and subjected to flexion–compression at a rate of 5 mm/s until the vertebral body was compressed more than 50 %, and lastly were followed by disruption of the posterior longitudinal ligament, ligamentum flavum, and supraspinous and interspinous ligaments with our intension to cause a 3-column injury to replicate a clinically relevant, challenging condition for fixation hardware to stabilize. Lateral radiographs and CT scans were routinely taken to demonstrate the extent of destabilization (Fig. 1a, b). The anterior body height [17] compression and load sharing classification score (LSC) [5] were used in describing the instability model, and the recovery rate of the anterior body height was calculated as follows: (preoperative compression rate-postoperative compression rate)/preoperative compression rate **×** 100 %, which were showed in Table 2.

**Table 1:** Sequence of conditions tested

Step	Condition
1	normal
2	after creating L1 burst fractures
3	after performing T12–L2 pedicle-screw fixation (SSPI) or T12–L1 pedicle-screw fixation (MSPI)
4	after fatigue cyclic loading

SSPI indicates short-segment pedicle instrumentation; MSPI indicates monosegmental pedicle instrumentation

**Table 2:** Roentgenographic data of the instability model between the two groups mean (standard deviation)

Parameter	Before fixation	After fixation	Recovering rate (%)
Load sharing Score			
SSPI	6.3 (1.8)	n/a	n/a
MSPI	6.6 (2.1)	n/a	n/a
FG in study 2	6.2 (2.3)	n/a	n/a
Anterior body height compression (%)			
SSPI	40 (11)	4.0 (1.0)	90.0 (11.1)
MSPI	41 (14)	3.0 (1.4)	92.7 (8.0)

SSPI indicates short-segment pedicle instrumentation; MSPI indicates monosegmental pedicle instrumentation; FG indicates fractured group

n/a indicates not available

Pedicle screws were inserted using standard techniques by a fellowship-trained, spine surgeon, who was familiar with pedicle screw placement. All the pedicle screws were inserted using a digital torque driver (accuracy ± 0.5 %, Cedar Digital Driver: DSD-4 M; Sugisaki Meter Company, Ltd, Ibaraki, Japan). The insertional torque was monitored continuously during insertion, and the highest torque during insertion was defined as the “maximal insertional torque”. The highest torque acquired during the final 360°revolution of the screw was defined as the “seating torque” [18]. Fluoroscopy was used during pedicle screw insertion to ensure adequate placement. We placed the same size screws (5 × 35 mm, general spine system, GSS) at the vertebral bodies followed by interconnection using appropriate-sized connectors between T12-L1 in the MSPI group and T12-L2 in the SSPI group (Fig. 1c, d).

**Fig. 2:**
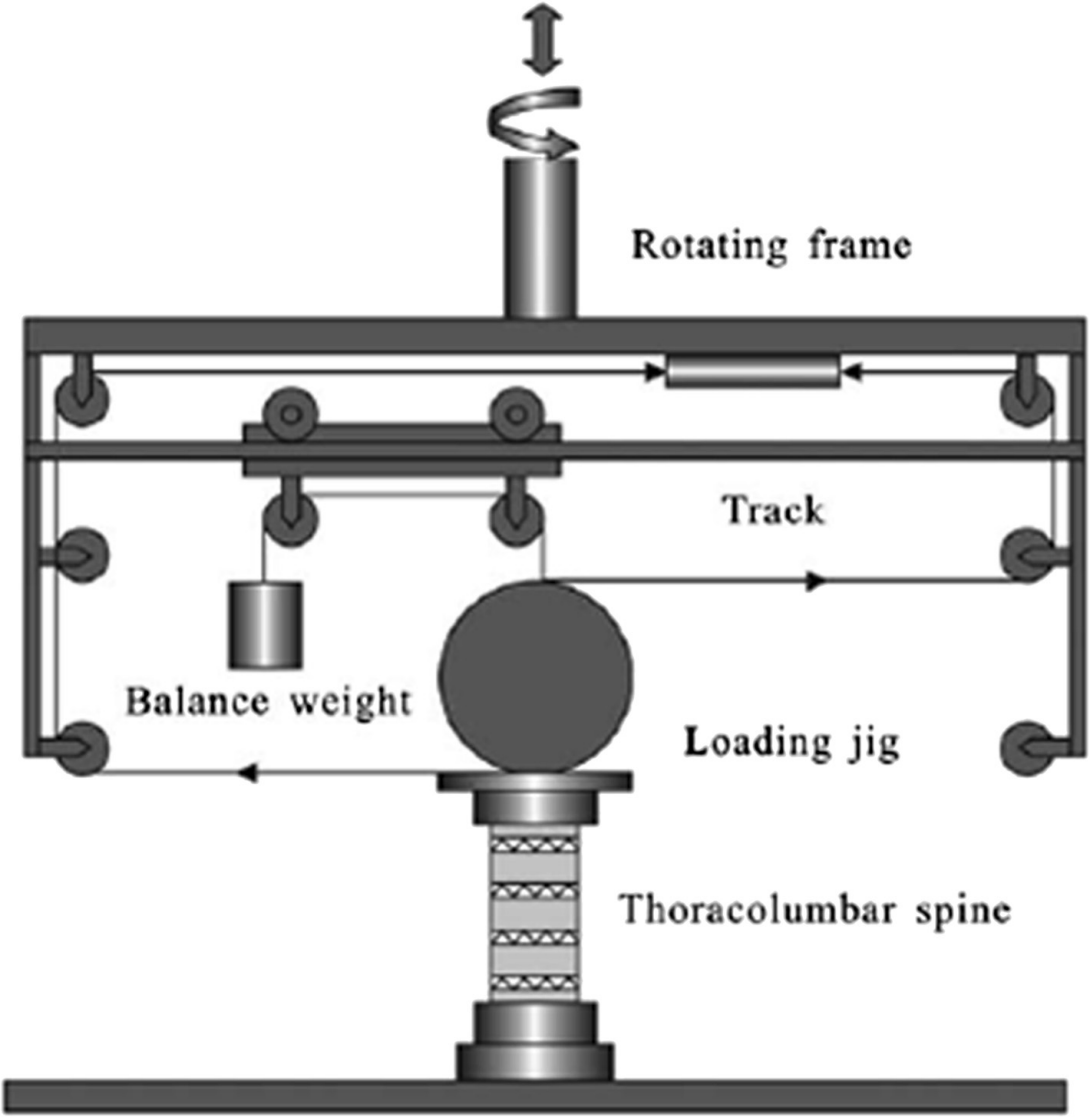
The diagram of the spine tester for three-dimensional spinal motion

**Fig. 3:**
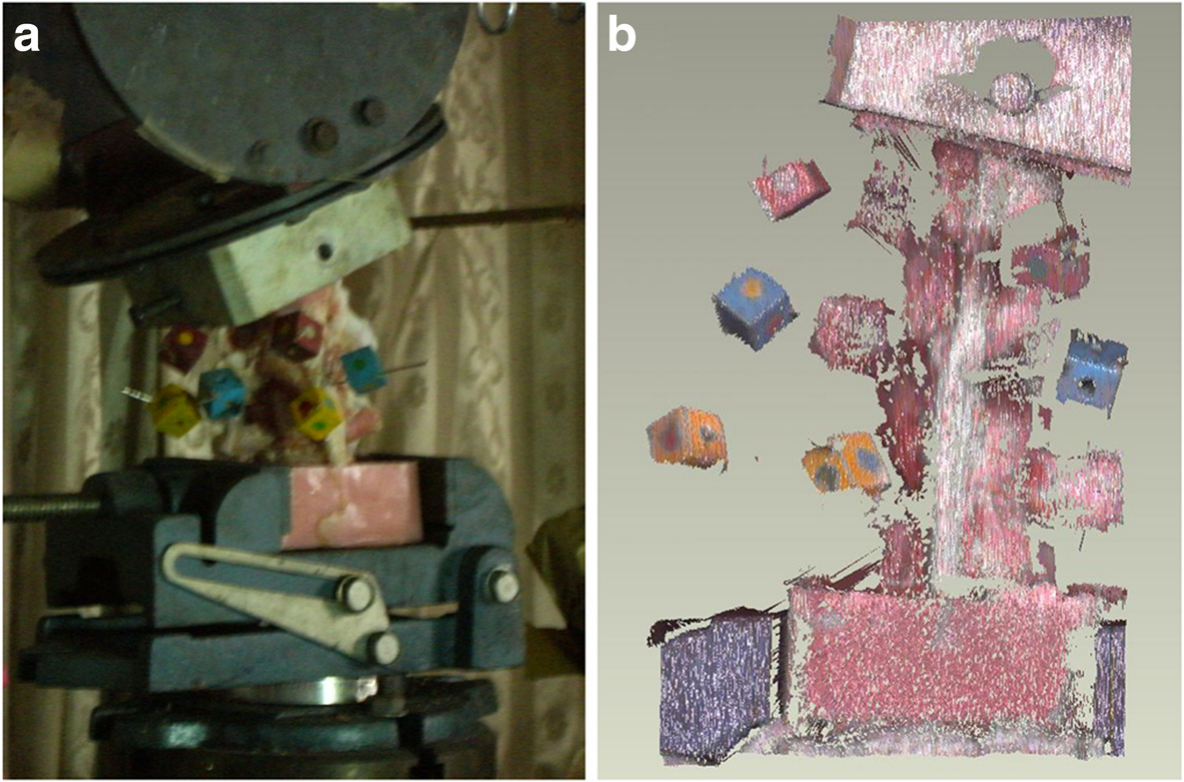
Calf specimen and stereo image. **a** Calf T12 to L2 specimen with three non-colinear circular marks in the spine tester. **b** Stereo image of the markers on the specimen were recorded by a 3-dimensional laser scanister

In each of the 4 conditions listed in Table 1, the specimen was mounted on a spine tester for three-dimensional spinal motion at room temperature (Fig. 2), as described previously [19]. The inferior cast was fixed to the machine, while the loading jig was attached to the superior cast (Fig. 3a). The machine applied six pure moments to the specimen in flexion, extension, bilateral bending, and bilateral axial rotation [19]. Each moment was applied in three load-unload cycles to a maximum 6 Nm and was allowed to creep for 30s at each load-step to account for viscoelastic effects [20, 21]. On the third load cycle, stereo images of the markers inserted into the transverse process and anterior vertebral body of the specimen were recorded by a 3-dimensional laser scanister (3D Digital Corp. United States, Fig. 3b) and stored on a computer. The marker coordinates were digitized using a previously developed computer program, and the motion capabilities of the whole specimen were determined in three-dimension. The accuracy of the measurement system has been determined [19]. The maximum error in marker position was 1.0 mm (1° in segmental angle) for a 60 mm × 60 mm × 150 mm measuring space. Throughout the tests, the specimens were kept wet using 0.9 % physiological saline.

Between the test sequence of step 3 and step 4, the specimens with pedicle instrumentation were fixed on the MTS material testing machine for the fatigue cyclic loading test. The fatigue parameter applied under torque control was a moment of ± 6 Nm conducted at a rate of 0.5Hz up to 2000 cycles of flexion-extension, lateral bending, and axial rotation respectively. To restore the physiologic hydration of the specimens, the specimens were wrapped in a saline-soaked gauze throughout the test.

From the raw data, the angular ROM across T12–L1 was calculated during motion in all planes. As an additional measure of stability, the angular lax zone (LZ, the portion of the ROM in which ligaments and hardware are not yet substantially loaded) was determined [22].

### Study 2

#### Specimen preparation

**Fig. 4:**
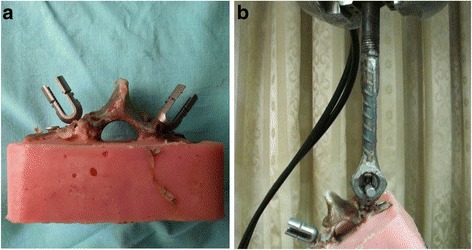
Pedicle screw pullout. **a** Samples embedded in PMMA. **b** The long axis of the screw was aligned to the axis of the machine to create a pure axial pullout force

Thirty-two fresh frozen vertebrae from 6 to 8 weeks old calf spines with all soft tissue removed (T11-L3) were randomized into two groups: intact group (IG) and fractured group (FG). The vertebral fracture models of the FG were created as described previously, and the load sharing classification scores (LSC) were recorded. The same size pedicle screws were inserted into the vertebral bodies of both groups by the same surgeon, and the “maximal insertional torque”, “seating torque” were again measured. Each specimen was then individually potted in a casting mold of PMMA, which encased the vertebral body, leaving the posterior elements and pedicle screws exposed (Fig. 4a).

#### Axial pullout test

**Fig. 5:**
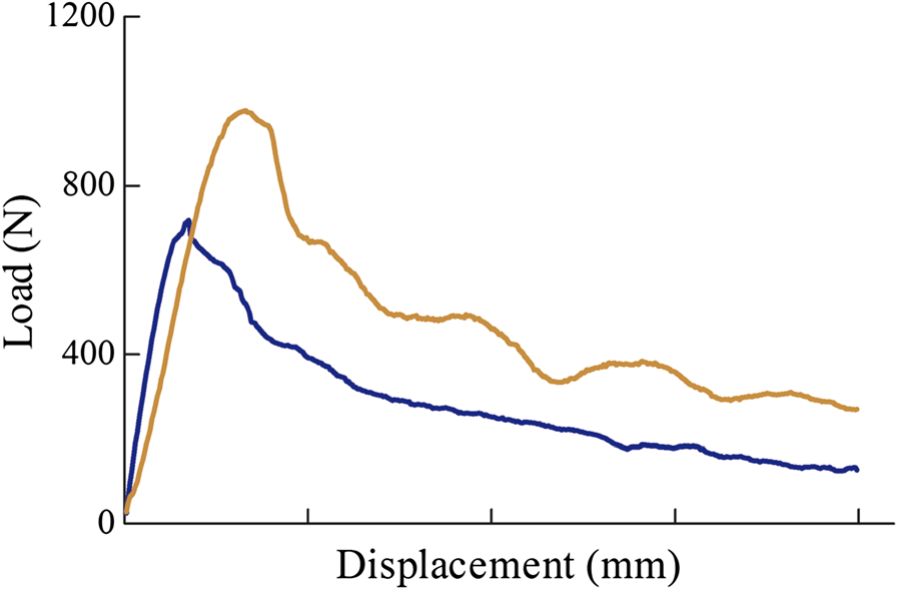
Two example load–displacement curves acquired from pullout testing. The pullout strength is determined at the maximum value of the curve

Axial pullout tests were conducted in the specimens using the MTS machine. In brief, the PMMA mold was rigidly secured to the base of the testing apparatus using an angle vise. The long axis of the screw was aligned to the axis of the machine to create a pure axial pullout force on each screw (Fig. 4b). Pull-to-failure tests were performed with a starting preload of 1 N and crosshead speed of 1 mm/s. Failure load was recorded for each pullout test (Fig. 5). The maximum force to pull the screw out from the pedicle was recorded as the axial pullout strength. The pullout strength of each vertebra was the mean of the sum of pullout strength calculated from both left and right side of pedicle screws.

#### Data analysis

The SPSS (version16.0, Chicago, IL, USA) package was used for the statistical analysis. The ROM and LZ of the same condition between groups were analyzed using Student’s *t*-test. The ROM and LZ at different conditions in each group were analyzed using one-way ANOVA and the least significant difference test (LSD-t). The “maximal insertional torque”, “seating torque” and the axial pullout strength between the normal and fractured vertebrae were compared using Student’s *t*-test, respectively. Pearson correlation coefficient was used to correlate the biomechanical data with LSC score of the specimens. Statistical significance was indicated at *P* < 0.05.

## Results

### Study 1

There were no significant differences of LSC and anterior body height compression rate between the instability models of the two groups (*P* > 0.05, see Table 2). There was also no significant difference in the recovery rate of the anterior body height compression between groups (*P* > 0.05).

**Fig. 6:**
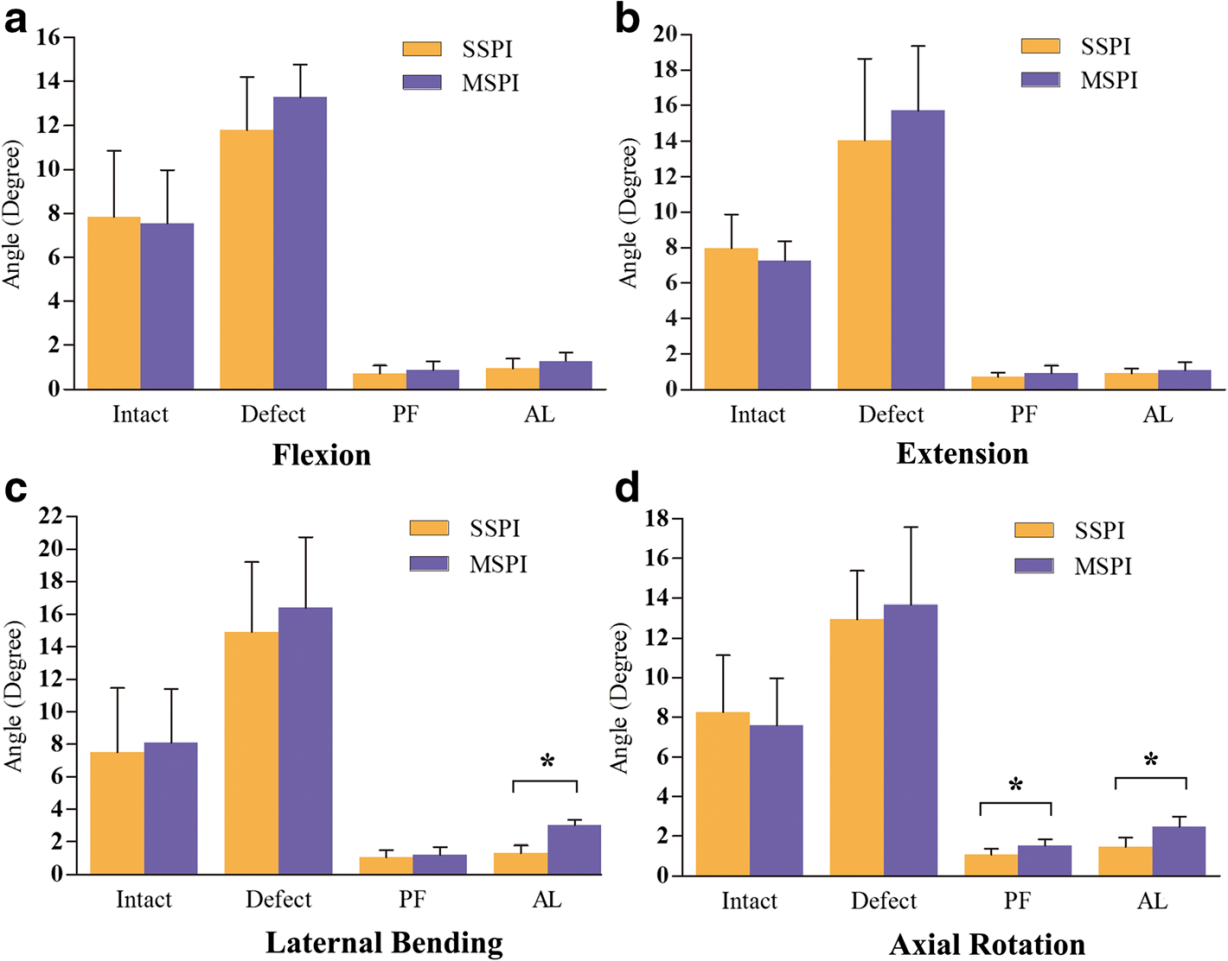
The mean angular ROM of T12–L1 for all loading directions. **a**. The mean angular ROM of T12–L1 in flexion. **b**. The mean angular ROM of T12–L1 in extension. **c**. The mean angular ROM of T12–L1 in lateral bending. **d**. The mean angular ROM of T12–L1 in axial rotation. Error bars show standard deviation. *: *P* < 0.05; ROM: range of motion; PF: pedicle fixation; AL: after cyclic loading

**Fig. 7:**
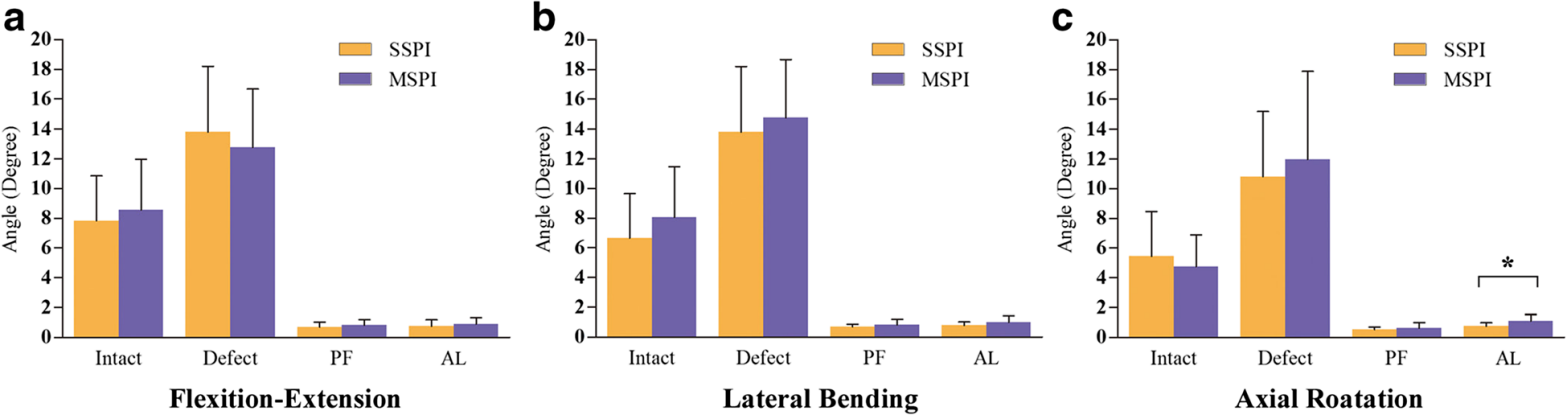
The mean angular LZ of T12–L1 for all loading directions. **a**.The mean angular LZ of T12–L1 in the direction of flexion-extension. **b**. The mean angular LZ of T12–L1 in the direction of lateral bending. **c**. The mean angular LZ of T12–L1 in the direction of axial rotation. Error bars show standard deviation. *: *P* < 0.05; LZ: lax zone; PF: pedicle fixation; AL: after cyclic loading.

Data for ROM from both right and left lateral bending and axial rotation showed no difference (*P* > 0.05) and were combined when used for comparison. For all three motion planes (lateral bending, flexion/extension, and axial rotation), the instability model showed an increased ROM and LZ when compared with the intact status in each group respectively (Fig. 6, Fig. 7, P < 0.01). Both of the SSPI and MSPI significantly reduced the ROM and LZ across T12–L1 to within the mean values observed in the intact condition (Fig. 6, Fig. 7, *P* < 0.05).

After stabilization, the SSPI allowed less ROM than MSPI during any loading model, however, except in the axial rotation, there were no significant differences in the other directions (Fig. 6, *P* > 0.05). Similarly, the SSPI allowed less LZ than MSPI during any loading model; however, there were no significant differences in all the directions (Fig. 7, *P* > 0.05).

After fatigue cyclic loading, although both groups allowed greater ROM and LZ than constructs during any loading model, there were no significant differences (*P* > 0.05), except in the LZ during axial rotation of the MSPI (*P* < 0.05). Although there were no significant differences in the LZ during all loading models between groups (*P* > 0.05), the MSPI group showed a significant increase in ROM than the SSPI group during the lateral bending and axial rotation after fatigue cyclic loading (Fig. 6, Fig. 7, *P* < 0.05).

### Study 2

#### Insertional torque

Both of the mean maximal insertional torque and seating torque in the fractured vertebrae decreased significantly compared to that in the intact vertebrae (*P* > 0.01, see Table 3). Although the mean axial pullout strength in the fractured vertebrae decreased compared to that in the intact vertebrae, the difference was not significant (*P* > 0.05, see Table 3).

**Table 3:** Performance of the screws in intact and fractured vertebrae: Mean (standard deviation)

Parameter	Fractured vertebrae	Intact vertebrae	% Decrease	*P* value
Maximal insertional torque (Nm)	0.29(0.13)	0.40(0.15)	27.5	0.001
Seating torque in (Nm)	0.23(0.11)	0.28(0.13)	17.9	0.04
Axial pullout strength (N)	558(305)	646(266)	13.7	0.06

#### Biomechanical data correlation with LSC

**Fig. 8:**
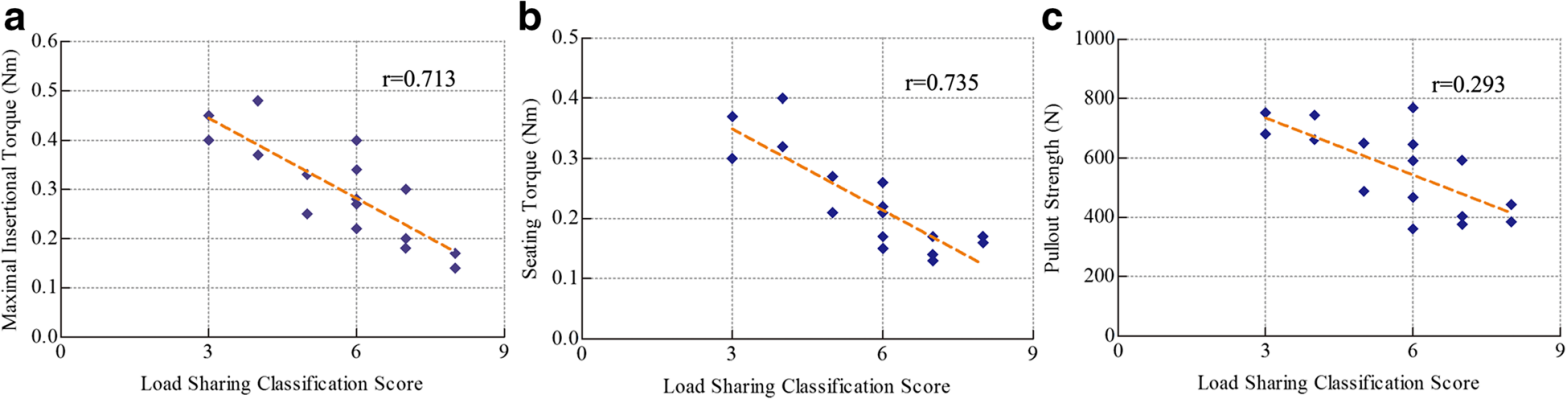
The correlation between maximum insertional torque, seating torque, pullout strength and the Load Sharing Classification (LSC) score of the fractured vertebrae. **a**. The correlation between the maximum insertional torque and the Load Sharing Classification (LSC) score of the fractured vertebrae. **b**. The correlation between the seating torque and the Load Sharing Classification (LSC) score of the fractured vertebrae. **c**. The correlation between the pullout strength and the Load Sharing Classification (LSC) score of the fractured vertebrae.The axial pullout strength had a low correlation with LSC of the specimens (r = 0.293, *P* > 0.05)

The mean load sharing classification score (LSC) of the specimens was 6.2 ± 2.3 points (see Table 2). The maximal insertional torque (*r* = 0.713) and seating torque (*r* = 0.735) for the screws had a high correlation with LSC of the specimens (*P* > 0.01, Fig. 8). However, the axial pullout strength (*r* = 0.293) had a low correlation with LSC of the specimens (*P* > 0.05, Fig. 8).

## Discussion

Although the surgical method to be selected in the treatment of thoracolumbar fractures remains a matter of discussion [1, 23], instrumented posterior fusion is the most frequently used surgical treatment because of the low morbidity and comorbidity, and surgeons’ familiarity with posterior approach compared with anterior approach. Short-segment pedicle instrumentation (SSPI), which typically span the levels immediately adjacent to the fracture, is almost the most widely used [24–26].

With the advent of minimal invasive surgery, an important consideration is achieving stability with fusion of the fewest number of motion segments. Some authors have been using monosegmental pedicle instrumentation (MSPI) to treat thoracolumbar fractures [3, 11, 27]. We also used this method in management of 47 patients with thoracolumbar fractures and compared the clinical and radiologic late results with that of short-segment pedicle instrumentation (SSPI). The results showed no significant differences in the correction rate of local kyphosis and anterior body height compression, and there were also no significant difference in the average correction loss of all radiographic parameters between the two methods [12]. However, the biomechanical characteristics of MSPI in management of unstable thoracolumbar fractures and whether the MSPI can provide effective immediate stability equivalent to that provided by traditional SSPI is still uncertain.

In this study, we found that both of the SSPI and MSPI significantly reduced the ROM and LZ across T12–L1 to within the mean values observed in the intact condition, which predicted that the MSPI could provide equal immediate stability to that of SSPI with respect to the reconstruction of unstable 3-column fractured models. However, in clinical applications, one of the most commonly observed internal failure is loosing of the pedicle screws due to fatigue cyclic loading. Therefore, this study also designed to measure the stability of the pedicle instrumentation after 2000 cyclic loading to represent the early situation of post-operation, as described in previous studies [28–30]. After cyclic loading, the ROM for the lateral bending and axial rotation in the MSPI increased significantly in comparison with the SSPI. The possible reason for this was shear forces of pedicle screw induced in the process of cyclic loading, which could affect the screw stability in the fractured vertebrae. Whatever the reason for the observed outcome, this predicted that the effectiveness of immediate fatigue resistance of MSPI is inferior to SSPI, especially in the lateral bending and axial rotation, which should be paid more attention to in the clinical application.

Previous studies have revealed that high incidence of instrumentation failure and loss of kyphotic correction after SSPI was caused mainly by the structural and mechanical deficiency of the anterior column after indirect reduction of the fracture [4, 31]. A general assessment system of fractured vertebral body, the load sharing score (LSC), is a successful way to predict clinically successful SSPI. Dai et al. [32] proved that the LSC can be applied with excellent reliability for assessing thoracolumbar fractures. Gaines et al. [33] made a conclusion from their clinical experiences that a LSC ≤ 6 indicated adequate sharing of load through the injured vertebral body along with SSPI, whereas a LSC ≥ 6 predicted highly possible failure of SSPI, and should be avoided. In this study, although the average LSC of the instability models in each group was more than 6, no significant increase of ROM after cyclic loading was found in both groups, except in the LZ during axial rotation of the MSPI. This suggested that thoracolumbar fractures with LSC ≥6 could be not a strict contraindication for MPSI. However, this need to be verified by further clinical study.

There are understandable concerns that the pullout strength of the pedicle screw inserted into the fractured vertebral body may not be sufficient for the stability reconstruction. It was illustrated that the pedicle, rather than the vertebral body, contributed ~60 % of the pull-out strength at the screw-bone interface [34]. Zindrick et al. [35] reported no difference in pullout strength between similar-size screws inserted to a 50 % depth versus to anterior cortex. So long as there is integrity of both pedicles, the screws inserted into the fractured level are supposed to have fairly tolerable stiffness and pullout strength. To further analyze the contribution of the fractured vertebrae to screw stability, axial pullout was carried out and compared for the screws inserted into the fractured vertebrae and normal vertebrae in this study. The maximal insertional torque and seating torque of screws inserted into the fractured vertebrae decreased significantly compared with that of intact vertebrae (*P* > 0.05), but not resulting in greater drop of axial pullout strength for the screws. The insertion torque is believed to result from resistance of the screw with bone and also from radial compression of the trabecular against cortical bone in the vertebral body [36]; however, the pedicle is denser in the subcortical bone, in which the threads of the screw engage tightly, than in trabecular bone. This may explain why the pullout strength of the pedicle screw is less affected by the vertebral fracture. This finding is also in agreement with data analyzed the correlation of the screw stability with LSC in the current study, where the strength of the correlation with LSC dropped in pullout strength of the screws (*r* = 0.293), compared with maximal insertional torque (*r* = 0.713) and seating torque (*r* = 0.735). Importantly, the axial pullout strength of screws inserted into the fractured vertebrae just dropped by 13.7 % of screws inserted into the normal vertebrae, which showed no significant difference. This result confirmed the previously reported conclusion that the pedicle was the major structure that contributed most of the pull-out strength at the screw-bone interface [34].

This experiment utilized calf spines. Despite the differences between calf and human anatomy [37], the elasticity and anatomical structure of calf spines are similar to human spines. As well, the difference in size is small, and there are no pathological changes, such as joint degeneration and osteoporosis in the calf spines. These characteristics are all useful when doing this experiment, and calf spine was regarded as the most suitable substitute material for the human spine [38]. However, although the unstable fracture model has been widely used in biomechanical experiments, the model does not take into account the stability effect of the neuromuscular structure on the spine. As well, in vitro experiments can only evaluate biomechanical changes during the early post-operative period, unlike clinical observations of the dynamic process of spinal fusion, during which intervertebral stability strengthens as the bone gradually heals. Therefore, designing a suitable model that can simulate the entire spine, as well as the mechanical and loading conditions, remains a challenge. In addition, due to laboratory facility limitations, this experiment did not investigate the rod strain of the instruments and the post-fixation stress of adjacent joints. Given our results, further clinical observation and biomechanical study is warranted.

## Conclusion

This current study demonstrated that MSPI can provide effective immediate stability for management of unstable thoracolumbar fractures, however, it has less fatigue resistance during lateral bending and axial rotation compared with SSPI. The axial pullout strength of screws inserted into the fractured vertebrae dropped by 13.7 % of screws inserted into the normal vertebrae. LSC score of fractured vertebrae is not a major influence of the pullout strength of screws.

## References

[1] Wood K, Buttermann G, Mehbod A, Garvey T, Jhanjee R, Sechriest V (2003). Operative compared with nonoperative treatment of a thoracolumbar burst fracture without neurological deficit. A prospective, randomized study. J Bone Joint Surg Am.

[2] Dick W, Kluger P, Magerl F, Woersdörfer O, Zäch G (1985). A new device for internal fixation of thoracolumbar and lumbar spine fractures: the ‘fixateur interne’. Paraplegia.

[3] Finkelstein JA, Wai EK, Jackson SS, Ahn H, Brighton-Knight M (2003). Single-level fixation of flexion distraction injuries. J Spinal Disord Tech.

[4] Gelb D, Ludwig S, Karp JE, Chung EH, Werner C, Kim T, Poelstra K. Successful treatment of thoracolumbar fractures with short-segment pedicle instrumentation. J Spinal Disord Tech. 2010;23(5):293–301. 10.1097/BSD.0b013e3181af20b6 20606547

[5] Aligizakis AC, Katonis PG, Sapkas G, Papagelopoulos PJ, Galanakis I, Hadjipavlou A (2003). Gertzbein and load sharing classifications for unstable thoracolumbar fractures. Clin Orthop Relat Res.

[6] Kose KC, Inanmaz ME, Isik C, Basar H, Caliskan I, Bal E (2014). Short segment pedicle screw instrumentation with an index level screw and cantilevered hyperlordotic reduction in the treatment of type-A fractures of the thoracolumbar spine. Bone Joint J.

[7] Scholl BM, Theiss SM, Kirkpatrick JS (2006). Short segment fixation of thoracolumbar burst fractures. Orthopedics.

[8] Yurac R, Marré B, Urzua A, Munjin M, Lecaros MA (2006). Residual mobility of instrumented and non-fused segments in thoracolumbar spine fractures. Eur Spine J.

[9] Liljenqvist U, Mommsen U (1995). Surgical treatment of thoracolumbar spinal fractures with internal fixator and transpedicular spongiosa-plasty. Unfallchirurgie.

[10] Defino HL, Scarparo P (2005). Fractures of thoracolumbar spine: monosegmental fixation. Injury.

[11] Liu S, Li H, Liang C, Long H, Yu B, Chen B, Han G, Zhang X, Li F, Wei F. Monosegmental transpedicular fixation for selected patients with thoracolumbar burst fractures. J Spinal Disord Tech. 2009;22(1):38–44. 10.1097/BSD.0b013e3181679ba3 19190433

[12] Wei FX, Liu SY, Liang CX, Li HM, Long HQ, Yu BS, Chen BL, Chen KB. Transpedicular fixation in management of thoracolumbar burst fractures monosegmental fixation versus short-segment instrumentation. Spine (Phila Pa 1976). 2010;35(15):E714–720. 10.1097/BRS.0b013e3181d7ad1d 20535041

[13] Wilke HJ, Geppert J, Kienle A (2011). Biomechanical in vitro evaluation of the complete porcine spine in comparison with data of the human spine. Eur Spine J.

[14] Wilke HJ, Wenger K, Claes L (1998). Testing criteria for spinal implants: recommendations for the standardization of in vitro stability testing of spinal implants. Eur Spine J.

[15] Lu WW, Luk KD, Ruan DK, Fei ZQ, Leong JC (1999). Stability of the whole lumbar spine after multilevel fenestration and discectomy. Spine (Phila Pa 1976).

[16] Mermelstein LE, McLain RF, Yerby SA (1998). Reinforcement of thoracolumbar burst fractures with calcium phosphate cement. A biomechanical study. Spine (Phila Pa 1976).

[17] Rommens PM, Weyns F, Van Calenbergh F, Goffin J, Broos PL (1995). Mechanical performance of the Dick internal fixator: a clinical study of 75 patients. Eur Spine J.

[18] Stauff MP, Freedman BA, Kim JH, Hamasaki T, Yoon ST, Hutton WC (2014). The effect of pedicle screw redirection after lateral wall breach--a biomechanical study using human lumbar vertebrae. Spine J.

[19] Zhu Q, Ouyang J, Lu W, Lu H, Li Z, Guo X, Zhong S. Traumatic instabilities of the cervical spine caused by high-speed axial compression in a human model. An in vitro biomechanical study. Spine (Phila Pa 1976). 1999;24(5):440–4. 10.1097/00007632-199903010-00006 10084180

[20] Panjabi MM (1988). Biomechanical evaluation of spinal fixation devices: I. A conceptual framework. Spine (Phila Pa 1976).

[21] Yamamoto I, Panjabi MM, Crisco T, Oxland T (1989). Three-dimensional movements of the whole lumbar spine and lumbosacral joint. Spine (Phila Pa 1976).

[22] Crawford NR, Peles JD, Dickman CA (1998). The spinal lax zone and neutral zone: measurement techniques and parameter comparisons. J Spinal Disord.

[23] Wood KB, Li W, Lebl DS, Ploumis A (2014). Management of thoracolumbar spine fractures. Spine J.

[24] Khare S, Sharma V (2013). Surgical outcome of posterior short segment trans-pedicle screw fixation for thoracolumbar fractures. J Orthop.

[25] Dai LY, Jiang LS, Jiang SD (2009). Posterior short-segment fixation with or without fusion for thoracolumbar burst fractures. a five to seven-year prospective randomized study. J Bone Joint Surg Am.

[26] Pellisé F, Barastegui D, Hernandez-Fernandez A, Barrera-Ochoa S, Bagó J, Issa-Benítez D, Cáceres E, Villanueva C. Viability and long-term survival of short-segment posterior fixation in thoracolumbar burst fractures. Spine J. In press. 10.1016/j.spinee.2014.03.012 24642054

[27] Junge A, Gotzen L, von Garrel T, Ziring E, Giannadakis K (1997). Monosegmental internal fixator instrumentation and fusion in treatment of fractures of the thoracolumbar spine. Indications, technique and results. Unfallchirurg.

[28] Yu BS, Zhuang XM, Zheng ZM, Zhang JF, Li ZM, Lu WW (2010). Biomechanical comparison of 4 fixation techniques of sacral pedicle screw in osteoporotic condition. J Spinal Disord Tech.

[29] Zheng ZM, Zhang KB, Zhang JF, Yu BS, Liu H, Zhuang XM (2009). The effect of screw length and bone cement augmentation on the fixation strength of iliac screws: a biomechanical study. J Spinal Disord Tech.

[30] Zhu Q, Lu WW, Holmes AD, Zheng Y, Zhong S, Leong JC (2000). The effects of cyclic loading on pull-out strength of sacral screw fixation: an in vitro biomechanical study. Spine (Phila Pa 1976).

[31] McLain RF (2006). The biomechanics of long versus short fixation for thoracolumbar spine fractures. Spine (Phila Pa 1976).

[32] Dai LY, Jin WJ (2005). Interobserver and intraobserver reliability in the load sharing classification of the assessment of thoracolumbar burst fractures. Spine (Phila Pa 1976).

[33] McCormack T, Karaikovic E, Gaines RW (1994). The load sharing classification of spine fractures. Spine (Phila Pa 1976).

[34] Suk SI, Lee CK, Kim WJ, Chung YJ, Park YB (1995). Segmental pedicle screw fixation in the treatment of thoracic idiopathic scoliosis. Spine (Phila Pa 1976).

[35] Zindrick MR (1991). The role of transpedicular fixation systems for stabilization of the lumbar spine. Orthop Clin North Am.

[36] Brasiliense LB, Lazaro BC, Reyes PM, Newcomb AG, Turner JL, Crandall DG, Crawford NR. Characteristics of immediate and fatigue strength of a dual-threaded pedicle screw in cadaveric spines. Spine J. 2013;13(8):947–56. 10.1016/j.spinee.2013.03.010 23602373

[37] Sheng SR, Wang XY, Xu HZ, Zhu GQ, Zhou YF (2010). Anatomy of large animal spines and its comparison to the human spine: a systematic review. Eur Spine J.

[38] Yu BS (2003). Biomechanical comparison of the posterolateral fusion and posterior lumbar interbody fusion using pedicle screw fixation system for unstable lumbar spine. Hokkaido Igaky Zasshi.

